# Development and psychometric characteristics of analog measures of parental empathy

**DOI:** 10.1371/journal.pone.0259522

**Published:** 2021-11-04

**Authors:** Samantha Gonzalez, Christina M. Rodriguez

**Affiliations:** Department of Psychology, University of Alabama at Birmingham, Birmingham, Alabama, United States of America; University of Colorado Boulder, UNITED STATES

## Abstract

Despite substantial literature on the effect of empathy on parenting, empathy research has historically suffered from definitional and methodological limitations. Parental empathy can be characterized as parents’ ability to recognize, take the perspective of, and appropriately react to children’s emotions. Current parental empathy assessment largely relies on self-report measures of dispositional empathy, but many argue parental empathy is distinct from dispositional empathy. Despite efforts to measure parental empathy implicitly, such analog approaches are labor intensive. The current report describes the preliminary development of the Empathy Measure for Parents Analog Task (EMPAT), two novel analog measures of parental empathy: one EMPAT analog uses audio stimuli and a second version uses written evocative scripts. After piloting with a sample of undergraduate students (Study 1), the measures were then administered to a sample of 212 parents (Study 2). For each study, the accuracy of the audio and script stimuli were first confirmed by examination of frequency distributions, then exploratory factor analyses were conducted to determine factor structure for each emotion subscale (i.e., Happy, Mad, Sad, Scared), and finally the composition of each emotion subscale was confirmed with scale reliability analyses. Correlations between each EMPAT version and measures of dispositional empathy, parental empathy, and positive parenting indicators were examined to assess the initial validity of the EMPAT measures. The new analog tasks demonstrated good reliability as well as preliminary evidence of validity, with potential utility in assessing cognitive elements of empathy in particular. With continued efforts to examine measure validity, the implications of these studies suggest the EMPAT tasks show promise in providing improved implicit, efficient assessments of child-directed empathy, which may be important for understanding positive and problematic parenting.

## Introduction

Empathy is an often-studied construct in the psychological literature, albeit nebulous due to the lack of a singular definition [1,2]. Research definitions of empathy span cognitive, affective, somatic, and behavioral domains, yet the most widely accepted definition of empathy with the best evidence includes both affective and cognitive dimensions [2–6]. Affective empathy requires an empathic person experience an emotional response appropriate to the situation and the emotional response of another (e.g., if someone is crying, an empathic person would feel sadness). In the current study, this appropriate affective response is termed *empathic concern*, indicating one is affectively influenced by the emotions of another [4,7,8]. References to cognitive aspects of empathy include one’s ability to recognize another’s emotion (*emotion identification* or *recognition*) and to understand another’s viewpoint (*perspective taking*) [4,9]. These two aspects of empathy (affective and cognitive empathy) are clearly intertwined—one’s ability to experience empathic concern is directly tied to one’s ability to recognize and appreciate the emotions of another person.

Importantly, these broad definitions of empathy have been applied to dispositional empathy, which is construed as one’s generalized ability to understand and express empathic concern for the emotions of others [10]. However, dispositional empathy may diverge from parental empathy. Parental empathy is distinct from dispositional empathy as it theoretically denotes a parent’s ability to accurately identify, understand and effectively express concern for the emotions of their child [11] rather than simply empathize with the emotional state of others broadly. In other words, a parent may not evidence strong dispositional empathy toward other people in general yet experience substantial empathy toward their own child.

Parents’ deficits in dispositional empathy have long been studied as a catalyst for increased risk of physical child abuse and parent-child aggression across childhood [12–16] with the greatest risk of child physical abuse apparent in the first year of life [17]. By contrast, stronger empathic abilities have been touted as a skill integral to positive parenting [18–20]. In order to optimize its relevance to various facets of parenting, research on parents’ empathy highlights the importance of assessing parents’ abilities to recognize and to understand children’s emotions and reactions to best meet the child’s needs [11,20]. Indeed, parents’ empathy both contributes to, and exemplifies, positive parenting [19], security of the parent-child attachment relationship [20,21], and parents’ observed sensitivity to their children’s emotional states [22]. Parents’ greater empathic abilities not only convey emotional attunement with children but are also associated with parents’ use of positive, non-punitive discipline tactics and negatively related to their use of corporal punishment [23]. Empathic parenting in turn facilitates several positive child outcomes including children’s development of better physiologic regulation [24], emotion regulation [21], attachment security [20,25], greater empathic abilities [26], and prosocial responding [26]. Parents’ empathy has clear implications for the safety and well-being of children long term; thus, the need for understanding and promoting parental empathy early in life is imperative to reduce abuse risk and increase adaptive child outcomes.

Despite the significance of empathy in the parenting literature, research relies largely on self-reported measures of dispositional empathy to approximate parents’ empathic abilities toward their children. Due, in part, to the definitional issues in empathy research, assessment quality of the construct has been uneven. Traditionally empathy has been measured via explicit self-report methods [3,8,9]. However, self-report measures demonstrate a variety of limitations, often relying on highly transparent questionnaire items, eliciting social desirability biases, expecting accurate personal insight into one’s own empathic abilities, and utilizing generic terms that leave the target of one’s empathy ambiguous. Although self-report measures of dispositional empathy are often used as proxies for parental empathy when applied to parent samples [12,15,27,28], or parents are instructed to envision their children when completing dispositional empathy measures [27], these approaches make a fundamental assumption that indicators of one’s dispositional empathy are equivalent to one’s parental empathy. To address the shortcomings of self-report assessments of parental empathy, previous research has attempted to examine parent specific empathy through coded parent interviews [e.g., 11,20,24,25,29]. Interview assessments of parental empathy demonstrate that elements of parental empathy include indices such as attention to children’s signals, parenting beliefs, child attributions [11], and accurate judgment of children’s emotions [24]. Such results indicate parental empathy may be distinct from dispositional empathy. Thus, parental empathy is a more appropriate and valuable construct than dispositional empathy to understand facets relevant to parenting. Because parental empathy is viewed as a highly desirable quality to cultivate, and because parents’ child-directed empathy may provide more insight into parenting than broad other-directed empathy, researchers have pressed for the development of parental empathy measures [11,20,27], which may distinguish between dispositional and parental empathy, reduce response bias associated with self-report, and limit the laborious time demands of conducting and coding lengthy parent interviews.

Some have attempted to measure empathy through indirect means via analog tasks to reduce social desirability and to address the transparency and self-awareness needed for self-report measures. *Analog tasks* are assessments that strive to measure a construct indirectly to avoid detection by the participant, using implicit strategies or behavioral simulations [30,31]. Such methods are valuable for multiple reasons: the target of measurement and scoring is less obvious and less susceptible to participant manipulation than self-report questionnaires, and analog tasks aim to act as a proxy for how one would respond if presented with an analogous scenario outside of the laboratory. Analog tasks previously used to measure dispositional empathy include measuring one’s ability to identify an emotion just by viewing a pair of eyes [32], using Implicit Association Test paradigms [33], and measuring the ability to detect subtle emotion cues in language [34]. Inducing emotional arousal is another strategy used to assess empathic abilities *in vivo* [35,36], which involves presenting emotionally charged evocative stimuli or instructions to imagine someone in a distressing scenario. Use of evocative stimuli has proven efficacious in inducing a range of desired affective responses in participants. Dispositional empathic characteristics have been assessed with written evocative scripts [37] and evocative video clips [38]. Scripts have also been used to assess parents’ emotional responses to children’s pain [39] and videos have been used to evoke sadness in both parents and children to code parent responses [19], but evocative scripts designed to measure parental empathy in particular are lacking. For assessment of parental empathy specifically, evaluation of parent responses to a range of child emotions is imperative as each emotional state may require a unique set of parent reactions to be appropriate and helpful to the child. Additionally, parent reactions to different child emotions may provide information about different constructs; for instance, parents’ appropriate, happy reactions to a child’s happy state may have more specific implications for parental sensitivity whereas parents’ appropriate, calm reaction to a child’s angry state may contribute to parents’ lower child abuse risk [see similar discussion, 24]. Prior efforts to assess parental empathy via analog tasks using video [40] or eye tracking [41] involve equipment and considerable preparation and coding procedures that can be costly or complex, similar to the cost and labor of coding interviews [11,20,24,25,29], potentially laborious for both researchers and participants. Because designing measures of parental empathy that are implicit, robust, cost-effective, and straightforward remains an important need for parenting researchers, the present study aimed to create novel analog measures of parents’ empathic abilities.

### Present study

The current investigation sought to develop novel analog measures of parental empathy which have the potential to reduce response bias in parenting research, act as an implicit screening tool of child abuse risk, inform the positive parenting literature by creating a proxy for real parenting situations, and potentially have clinical utility as a training tool for improvement of parental empathy skills. Two versions (using audio and script stimuli) of a novel analog measure of parental empathy were developed in the current investigation. Both were piloted in an initial study with university students (Study 1) and revised versions of each were validated with parents in a second study (Study 2). The aim of the initial study (Study 1) was to assess whether emotion audio and script stimuli were performing as expected in the analog tasks as observed by emotion recognition in correctly identifying the target emotion for each stimulus. Additional goals of Study 1 were to establish reliability and evaluate validity, examining the association between the analog tasks and self-report measures of dispositional empathy. The aim of the subsequent study (Study 2) was to test the efficacy of the analog tasks in a parent sample following appropriate modifications gleaned from Study 1. The strength of the analog tasks was assessed with respect to evidence of internal consistency, convergent validity with parents’ self-reported parental empathy and dispositional empathy, and concurrent validity with measures of positive parenting.

## Study 1

The aim of this initial study was to pilot the two versions of the analog task with a sample of childless undergraduate students.

### EMPAT measure development

#### Empathy Measure for Parents Analog Task—Emotion Audio (EMPAT-EA)

The EMPAT-EA version was created by selecting two commercially available audio clips of children (e.g., from online sources Pond5, Envato, Stock Music) that were digitally remastered to play for 20 seconds for each of four target emotions: Happy, Mad, Sad, Scared. Each target emotion was represented by one non-verbal audio clip of a young boy expressing the emotion and one audio clip depicting a young girl expressing the emotion (e.g., sad represented by crying, happy by laughter, scared by shaky breathing, mad by tantrum/screaming), which resulted in a total of eight randomly presented 20-second audio clips.

Participants respond to two types of questions. Immediately following each clip, participants first report on how listening to the audio made them feel (i.e., “Self” questions), using a 5-point Likert scale for each of nine emotion items modeled after the Positive and Negative Affect Schedule (PANAS) [42] (0 = *very slightly or not at all*, 1 = *a little*, 2 = *moderately*, 3 = *quite a bit*, 4 = *extremely*). In this initial study, participants provided ratings for how much they felt each of these emotions while listening to each clip in the following order: Happy, Sad, Mad, Scared, Calm, Irritated, Worried, Cheerful, and Unhappy. These EMPAT-EA Self responses were theorized to reflect affective aspects of the empathy process (e.g., empathic concern). For the second type of questions, the audio files were re-played later in the protocol. This time after listening to each clip, participants were asked to indicate what they believed the child in the audio felt (i.e., “Child” questions); participants used the same 5-point Likert scale and rated the same set of nine emotions after every clip. EMPAT-EA Child responses were theorized to conceptually include cognitive aspects of the empathy process (e.g., emotion recognition).

#### Empathy Measure for Parents Analog Task—Emotion Scripts (EMPAT-ES)

Initial development of evocative scripts for the EMPAT-ES version began with a previous sample of parents (*N* = 183) of children aged 10 and under recruited through Amazon’s Mechanical Turk (MTurk). Parents in the MTurk study responded to four open-ended prompts to describe scenarios that would elicit each of four target emotions in their child (i.e., Happy, Mad, Sad, Scared). Common themes across all parent responses were identified, with each parent-provided scenario categorized into one of the themes (ranging from 32–37 different themes per emotion), and the four most frequently reported themes were identified for each target emotion. Based on each of the four themes, eight research assistants were instructed to independently generate four sample scripts that would elicit each target emotion in a four-year-old child. A final pool of 75 unique emotion scripts of two sentences each were created for the four target emotions, and then ranked for evocativeness by lab personnel (i.e., undergraduate and graduate psychology students) (e.g., “Rank each script based on which best captured the emotion the child in the scenario would most likely feel”). Finally, the three emotion script scenarios with the highest rankings overall for each target emotion were selected for inclusion in this pilot test of the EMPAT-ES analog task. Happy scenes included: finding a surprise birthday present; playing with a parent on a trampoline; and storytime with a parent. Scared scenes included: storm at night; feeling a bug crawling on them in the dark; and losing a parent in a crowd. Mad scenes included: parent revoking a child’s play-date; sibling stealing a favorite toy; and parent refusing their child candy at a store. Sad scenes included: death of grandmother; being left for the first time at daycare; and social exclusion at school. The final twelve emotion scripts were randomly presented, balanced by gender, and phrased to maintain a reading level no higher than 8^th^ grade.

In Study 1, participants were first asked to report on how reading each emotion script made them feel (i.e., “Self” questions) on a 5-point Likert scale (0 = *very slightly or not at all*, 1 = *a little*, 2 = *moderately*, 3 = *quite a bit*, 4 = *extremely*) for the same emotion items as the audio version: Happy, Sad, Mad, Scared, Calm, Irritated, Worried, Cheerful, and Unhappy. Responses to EMPAT-ES Self questions were theorized to conceptually involve affective aspects of empathy (e.g., empathic concern). Later in the protocol, participants rated how much the child in the evocative script (i.e., “Child”) felt each of these same emotion response items. EMPAT-ES Child responses were theorized to conceptually tap cognitive aspects of empathy (e.g., emotion recognition, perspective taking).

#### Scoring approach

Participants’ EMPAT-EA and EMPAT-ES Child scores about how the child in the analog stimuli felt would confirm the emotion classification accuracy of the stimuli and theoretically signify emotion recognition and perspective taking abilities (i.e., indicators of cognitive empathy) for both analog tasks.

Participants’ EMPAT-EA and EMPAT-ES Self scores were expected to include responses to questions about their own emotional state. Based on exploratory factor analyses (see Analytic Plan), responses contributing to each emotion subscale were identified. Using these analyses, four emotion subscale scores were calculated by averaging responses of the items for each of the Child and Self versions of both analog tasks (EMPAT-EA and EMPAT-ES), with primary interest being the composite scores that combine all four emotion subscales into total scores.

### Participants

Participants were 142 childless undergraduate students (*M*_age_ = 18.77 years, *SD* = 1.56; 59% female) enrolled in an Introductory Psychology course at a public urban university in the southeast U.S. Race was self-identified as: 65.5% White, 23.2% African American, 10.6% Asian, and 0.7% Native American/Alaskan Native; of these, 5.6% of participants identified as Hispanic and 15.5% as biracial. Undergraduates reported household incomes in their families of origin: 25% reported below $40,000, with a median between $60,000-$79,999.

### Dispositional empathy validity measures

#### Empathy quotient-short

The EQS [43] is a 22-item self-report measure of dispositional empathy. Participants indicated their level of agreement on a 4-point Likert scale on items suggestive of empathic abilities (e.g., “It is hard for me to see why some things upset people so much”). Approximately half of the items are reverse-scored, and a total score was computed by summing across items, with higher scores indicative of greater empathy. The test authors report good internal consistency (α = .90) and convergent validity. Good internal consistency (α = .88) was also observed in the current sample.

#### Interpersonal reactivity index

The IRI [44] is a 28-item measure of dispositional empathy. The IRI includes two relevant subscales of Empathic Concern (e.g., “Other people’s misfortunes do not usually disturb me a great deal”) and Perspective Taking (e.g., “Before criticizing somebody, I try to imagine how I would feel if I were in their place”), which were selected to assess participants’ ability to affectively empathize with and adopt the perspective of others, respectively. Each subscale is composed of seven items ranging from 1 (*does not describe me well)* to 5 (*describes me well)*. Subscale items were summed separately to create subscale scores, with higher scores reflecting greater empathy. The IRI evidences validity with comparable measures of empathy [44] and with measures of aggressive behavior [45]. Further, the IRI Empathic Concern and Perspective Taking subscales demonstrated acceptable internal consistency in the current study (α = .77 and .80, respectively).

### Procedures

Undergraduate students under the age of 26 were recruited for the current study (childless in order to homogenize the sample to avoid potential confounds from enrolling atypical undergraduates). Participants attended a 90-minute research session in small groups of six participants or fewer as an option to fulfill a research requirement in an introductory psychology course. All measures were delivered using Inquisit 4.0 software on individual desktop computers with headphones. After providing informed consent, the EMPAT-EA and EMPAT-ES Self analog tasks were administered at the start of the protocol followed by self-report measures of dispositional empathy and additional, unrelated measures, ending the session with the EMPAT-ES and EMPAT-EA Child versions. All study procedures were approved by the University of Alabama at Birmingham Institutional Review Board (IRB- 300000331).

### Analytic plan

Principal components exploratory factor analysis (EFA) and scale reliability analysis (Cronbach’s alpha) were used to determine the factor structure and reliability of each of four emotion subscales (i.e., Happy, Mad, Sad, Scared) for both the audio and script versions of the EMPAT analog measure. The EFAs included participants’ responses on the nine emotions (each item on a 5-point scale) provided for the Boy and Girl responses on each subscale (i.e., audio stimuli included 18 responses/subscale, script stimuli included 27 responses/subscale). We utilized Promax rotation of factors as needed assuming that any underlying factors would be non-orthogonal. We set a number of *a priori* criteria for item inclusion in the final subscale scores (guided by recommendations from Nunnally & Bernstein [46]). Items in the EFA needed to load at minimum .40 for subscale inclusion (those below this criterion were removed), and the variance explained by the remaining subscale items needed to account for at least 50% of the variance in the emotion subscale. Subscale reliability analyses then confirmed these final items contributed to the subscale, with minimum criteria set for alpha at least .70 and item-total correlations (ITCs) of at least .30. This set of analyses strived to empirically identify the most parsimonious factor structure of item sets to average in creating each of the four emotion subscale scores for each EMPAT version, with Total scores combining these averaged subscales.

### Results

#### Confirmation of stimuli accuracy

The accuracy of the emotions in the stimuli was confirmed using ratings from the EMPAT child versions. For the EMPAT-EA audio stimuli, 95% and 97.2% of respondents rated the Boy and Girl Happy audios, respectively, as “extremely” or “quite a bit” happy. The EMPAT-EA Boy and Girl Mad audios were rated as “extremely” or “quite a bit” mad by 86.7% and 86.6% of respondents, respectively. EMPAT-EA Sad Boy and Girl audio stimuli were correctly identified by 73.3% and 92.3%, respectively. The EMPAT-EA Scared audio stimuli were correctly identified in the Child version as Scared by 73.2% and 73.3% of respondents for the boy and girl audio, respectively. For the EMPAT-ES script stimuli, 93.6%, 94.4%, and 89.4% of respondents rated the three Happy scripts as “extremely” or “quite a bit” happy. The EMPAT-ES Mad scripts were rated as “extremely” or “quite a bit” mad by 83.1%, 79.6%, and 74.8% of respondents. The EMPAT-ES Sad scripts were correctly identified as sad by 94.4%, 84.6%, and 90.9% of respondents. The EMPAT-ES Scared scripts were rated as “extremely” or “quite a bit” scared by 89.5%, 91.6%, and 88.1% of respondents.

#### Factor analysis

See Table 1 for the final items included in each subscale. Across versions, one to two factors were ultimately identified. For the audio stimuli, the EMPAT-EA Child Happy subscale was comprised of 16 items (8 emotions each for Boy/Girl) and accounted for 83% of the variance. The Mad subscale included 6 items (3 emotions) that explained 60.95% of the variance. The Sad factor was comprised of 6 items (3 emotions) that explained 66.17% of the variance. The Scared factor consisted of 6 items (3 emotions) that accounted for 72.35% of the variance. For the EMPAT-EA Self audio stimuli, the Happy subscale was comprised of 6 items (3 emotions) that explained 62.68% of the variance. The Mad factor included 6 items (3 emotions) that explained 60.05% of the variance. The Sad factor was comprised of 8 items (4 emotions) explaining 53.13% of the variance. The Scared factor was comprised of 8 items (4 emotions) that explained 62.73% of the variance.

**Table 1:** Study 1: Emotion subscale scoring approach based on exploratory factor analyses (with EFA factor loadings for boy/girl versions of the stimuli).

	Study 1: Emotion Subscale Items
**EMPAT-EA Child**	
Happy	Happy (.82, .81), Cheerful (.84, .82), Sad*(.99, .99), Mad* (.93, .99), Scared* (.99, .99), Irritated* (.85, .85), Worried* (.97, .99), Unhappy* (.81, .74)
Mad	Mad (.83, .52), Irritated (.86, .57), Unhappy (.79, .84)
Sad	Sad (.70, .86), Unhappy (.64, .82), Worried (.91, .88)
Scared	Scared (.83, .81), Worried (.84, .80), Unhappy (.87, .93)
**EMPAT-EA Self**	
Happy	Happy (.76, .99), Cheerful (.80, .98), Calm (.98, .52)
Mad	Sad (.79, .81), Worried (.79, .78), Unhappy (.73, .75)
Sad	Sad, (.66, .78) Worried (.78, .77), Unhappy (.72, .75), Scared (.70, .67)
Scared	Sad (.78, .76), Scared (.76, .78), Worried (.81, .86), Unhappy (.80, .78)
**EMPAT-ES Child**	
Happy	Happy (.77, .80, .83), Cheerful (.83, .77, .68), Sad* (.87, .70, .82), Mad* (.95, .89, .82), Scared* (.88, .56, .81), Irritated* (.91, .83, .84), Worried* (.80, .69, .75), Unhappy* (.80, .79, .71)
Mad	Sad (.58, .82, .87), Mad (.78, .78, .73), Irritated (.89, .75, .71), Unhappy (.76, .70, .71)
Sad	Sad (.85, .83, .82), Unhappy (.78, .78, .77), Happy* (.96, .86, .97), Cheerful* (.92, .91, .98)
Scared	Scared (.82, .85, .75), Worried (.82, .79, .88), Unhappy (.81, .90, .78)
**EMPAT-ES Self**	
Happy	Happy (.82, .84, .93), Cheerful (.79, .81, .84), Calm (.90, .88, .62)
Mad	Mad (.65, .60, .84), Irritated (.78, .59, .97), Sad (.74, .80, .76), Unhappy (.57, .48, .71), Worried (.65, .50, .42)
Sad	Sad (.75, .77, .80), Worried (.67, .69, .67), Unhappy (.71, .69, .75)
Scared	Sad (.89, .92, .59), Scared (.82, .97, .68), Worried (.74, .89, .68), Unhappy (.44, .57, .54)

*Note*. Emotion words on the right indicate responses that met minimum criteria in the exploratory factor analysis for the respective emotion factor and were then included in the corresponding EMPAT emotion subscale average score.

*indicates the response was reverse scored.

For the EMPAT-ES Child script stimuli, the Happy subscale consisted of 24 items (8 emotions) that explained 66.53% of the variance. The Mad subscale was comprised of 12 items (4 emotions) accounting for 59.23% of the variance. The Sad subscale consisted of 12 items (4 emotions) that explained 76.21% of the variance. The Scared subscale was comprised of 9 items (3 emotions) that explained 68.87% of the variance. For the EMPAT-ES Self script stimuli, the Happy subscale included 9 items (3 emotions) that explained 70.37% of the variance. The Mad subscale consisted of 15 responses (5 emotions) that explained 51.42% of the variance. The Sad factor included 9 responses (3 emotions) that accounted for 65.59% of the variance. The Scared subscale included 8 items (4 emotions) that explained 61.58% of the variance.

#### Reliability

See Table 2 for scale alphas. For EMPAT-EA Child audio stimuli, the Total score demonstrated good reliability; all constituent emotion subscales (i.e., Happy, Mad, Sad, Scared) met the minimum criteria for alpha of at least .70. Examination of ITCs on emotion subscales confirmed that Happy (.32-.86), Sad (.47-.55), and Scared (.38-.69) all met the minimum criteria of at least .30. Although the ITC range for the EMPAT-EA Child Mad subscale fell just below this cutoff (.29-.57), the removal of that single item did not affect alpha; thus, the response was retained given that its comparable item in the other gender met the ITC criteria. For EMPAT-EA Self audio stimuli, all emotion subscales demonstrated high reliability and high ITCs in excess of the minimum criteria EMPAT-EA Self Happy (.57-.76), Mad (.61-.70), Sad (.55-.70), and Scared (.68-.80) subscales.

**Table 2:** Study 1: Means (standard deviations), reliability and validity of the EMPAT in Study 1.

	M(SD)	α	IRI_PT_ *r*	IRI_EC_ *r*	EQS *r*
**Audio Self**					
Happy	3.57 (0.92)	.88	.12	.31***	.14
Mad	3.20 (1.09)	.87	.16	.28***	.20*
Sad	2.76 (0.90)	.87	.14	.23**	.20*
Scared	3.15 (1.06)	.91	.18*	.28***	.26**
Total	12.68 (3.37)	.95	.18*	.33***	.24**
**Audio Child**					
Happy	4.89 (0.32)	.91	.14	.20*	.08
Mad	4.24 (0.66)	.71	.05	.04	.14
Sad	3.75 (0.77)	.75	.02	.16	.20*
Scared	3.90 (0.88)	.81	.13	.20*	.26**
Total	16.78 (2.05)	.88	.11	.20*	.24**
**Script Self**					
Happy	3.66 (0.85)	.90	.17*	.32***	.24**
Mad	2.24 (0.67)	.89	-.05	.03	.06
Sad	3.31 (0.89)	.88	.11	.21*	.19*
Scared	2.83 (0.88)	.91	.10	.23**	.13
Total (w/o Mad)	9.79 (2.32)	.95	.14	.29***	.21*
**Script Child**					
Happy	4.78 (0.43)	.92	.14	.18*	.13
Mad	3.72 (0.82)	.90	.19*	.22**	.23**
Sad	4.63 (0.45)	.81	.20*	.26**	.23**
Scared	4.19 (0.82)	.89	.23**	.30***	.24**
Total	17.32 (2.16)	.95	.23**	.29***	.25**
***M*(*SD*)**		25.79 (5.11)		27.13 (5.19)	67.45 (8.69)
**α**		.80		.77	.85

*Note*. IRI_EC_ = Interpersonal Reactivity Index, Empathic Concern subscale; IRI_PT_ = Perspective Taking subscale; IRI_T_ = Total Score. EQS = Emotion Quotient Short.

* p ≤ .05,

** p ≤ .01,

***p ≤ .001.

For the EMPAT-ES Child script stimuli, the Total score also demonstrated strong reliability; all emotion subscales also demonstrated strong internal consistency. Additionally, the Happy (.33-.86), Mad (.34-.72), Sad (.34-.61), and Scared (.55-.73) emotion subscales all exceeded the minimum criteria for ITCs. For the EMPAT-ES Self script stimuli, the Total score demonstrated excellent reliability as well as its emotion subscales. The EMPAT-ES Self Happy (.48-.77), Mad (.32-.70), Sad (.57-.69), and Scared (.47-.75) subscales also all met the minimum ITCs criteria.

#### Validity

See Table 2 for correlations. Using the audio stimuli, the EMPAT-EA Self Total score, and all emotion subscale scores, were significantly associated with dispositional empathic concern (i.e., affective empathy) on the IRI, demonstrating convergent validity. Only the EMPAT-EA Self Total score and the Scared emotion subscale score were significantly related to dispositional perspective taking (i.e., cognitive empathy) on the IRI. The EMPAT-EA Self Total score and all emotion subscales, except for Happy, were related to dispositional empathy measured with the EQS. The EMPAT-EA Child Total score, along with the Happy and Scared subscales, were significantly related to dispositional empathic concern on the IRI. No EMPAT-EA Child subscales were significantly related to dispositional perspective taking. The EMPAT-EA Child Total score, Sad subscale, and Scared subscale score were significantly associated with the EQS.

Regarding the script analog, the EMPAT-ES Self Total score and the Happy and Sad subscale scores were significantly associated with dispositional empathy on the EQS (see Table 2). All EMPAT-ES Self subscale scores, except for EMPAT-ES Self Mad, were significantly related to dispositional empathic concern on the IRI. Only the EMPAT-ES Self Happy subscale score was significantly associated with dispositional perspective taking on the IRI. Notably, the EMPAT-ES Self Mad subscale score was negative and not significantly related to any dimensions of dispositional empathy. This result suggests that participants’ reactions to a mad child did not provide useful information about their empathic skills, which ran counter to study expectations that reactions to child angry stimuli would elicit important information about empathic responding. Further, EMPAT-ES Self Mad also did not contribute to the scale reliability analysis for the EMPAT-ES Self Total score in the expected direction. Taken together, these results suggested that the Mad subscale was not performing as accurately as the other emotion subscales; thus, the Mad subscale was removed from the EMPAT-ES Self Total score for these analyses.

Most EMPAT-ES Child Total score and emotion subscale scores were significantly related to measures of dispositional affective and cognitive empathy on the IRI. All emotion subscale scores demonstrated significant associations with dispositional empathic concern (i.e., affective empathy) on the IRI. All emotion subscales except for EMPAT-ES Child Happy scores were significantly related to dispositional perspective taking (i.e., cognitive empathy) on the IRI. The Total EMPAT-ES Child scores and all subscale scores except EMPAT-ES Child Happy were significantly associated with total dispositional empathy scores on the EQS.

### Discussion

The goals for development of both the Self and Child versions of the EMPAT task in this pilot were three-fold: for participants to be able to accurately identify the child’s emotion (“Child” versions), experience empathic concern for the child (“Self” versions) and demonstrate understanding of the child’s perspective (“Child” versions). Findings from this initial study suggest that for the EMPAT-EA (using audio stimuli), Self responses were largely related to affective empathy (empathic concern), as expected. However, for the Child responses, although the total score was related to dispositional empathy, only some of the emotion scores appeared to contribute to that relation (EMPAT-EA Child Happy and Scared for the IRI and EMPAT-EA Child Sad and Scared for the EQS). Further, EMPAT-EA Child scores were minimally associated with aspects of perspective taking on the IRI, which would have been indicative of cognitive empathic abilities. These results contradict the expectation that EMPAT-EA Child responses would primarily be related to aspects of cognitive empathy (i.e., emotion recognition and dispositional perspective taking). Thus, fewer associations with self-reported dispositional perspective taking were observed, suggesting that neither Self nor Child versions of the EMPAT-EA may tap into cognitive empathy as expected. Alternatively, although the EMPAT-EA Self responses were expected to represent affective empathy and the EMPAT-EA Child responses were expected to represent cognitive empathy, the possibility remains that the two versions of the audio analog task may not be able to clearly distinguish affective from cognitive empathy.

Using evocative scripts, the EMPAT-ES Self responses provided some evidence of empathic concern as expected. However, the EMPAT-ES Self Mad subscale did not operate as expected and was not significantly related to any measure of dispositional empathy, suggesting that how individuals feel when reading about a situation that might anger a child did not evoke an emotional response reflected in empathic abilities. Individuals’ behavioral responses to children’s anger may be a better marker of empathy in the context of reacting to potentially frustrating child emotions. However, the inability of EMPAT-ES Self Mad scores to contribute to the primary EMPAT-ES Total score is concerning given that reactions to child anger in particular are critical to the principal goal of developing an empathy analog task—to capture parents’ reactions to multiple children’s emotions. The EMPAT-ES Child script version was expected to represent primarily cognitive aspects of empathy (e.g., emotion recognition, perspective taking) but results indicated that the script version was significantly related to both cognitive and affective aspects of dispositional empathy, indicating that the script analog may not differentiate these components of empathy.

Although Child responses on both EMPAT audio and script versions were expected to convey cognitive empathy (i.e., emotion recognition, perspective taking), the EMPAT-EA Child using audio stimuli demonstrated fewer significant associations with measures of dispositional empathy relative to the script version. This finding suggests EMPAT-ES evocative scripts may serve as a stronger measure of cognitive empathy. Self responses on both versions of the EMPAT evidenced associations with affective empathy (i.e., empathic concern), with the notable exception of EMPAT-ES Mad as noted above. However, overall, dispositional affective empathy was related to Child and Self versions of the EMPAT analog tasks rather than simply with the Self responses.

To further improve the measures for the next study with parents, in the EMPAT-EA, the emotion audio stimuli for Boy Sad, Boy Scared, and Girl Scared were all replaced with new audio clips because frequency distribution results showed that less than 75% of participants accurately identified these target emotions from the respective audio clips. In addition, for both the EMPAT-EA and EMPAT-ES, the “calm” response option did not load meaningfully on any of the target emotion subscales (originally intended for the Mad stimuli) and thus was eliminated as an item in the subsequent study. Similarly, the “unhappy” item appeared to function as anything other than “happy” (selected in nearly all emotion subscales), rather than as the intended synonym for “sad”. Thus, in the subsequent study, “unhappy” was replaced with “blue” to function as a brief, clearer synonym to “sad” in all versions. For emotion identification options (i.e., Child versions), to provide participants more items, “afraid” and “angry” were added. Finally, given the pilot finding that EMPAT-ES Self Mad did not operate as expected, nor in the same direction as the other three EMPAT-ES Self emotion subscales, the subsequent study only presented EMPAT-ES Child questions; omitting EMPAT-ES Self also reduced the protocol length for the next study. To further reduce the protocol length, the two strongest EMPAT-ES scripts for each emotion (i.e., the two with the highest correct emotion identification on EMPAT-ES Child as demonstrated by frequency distributions) were extracted for use in the next study. Taken together, these modifications were expected to improve the performance of both the EMPAT-EA and EMPAT-ES in measurement of emotion recognition (i.e., cognitive empathy) and empathic concern abilities (i.e., affective empathy) with the analog tasks’ target population—parents.

## Study 2

The goal of the second study was to assess the reliability and provide preliminary concurrent and convergent validity of the modified EMPAT with a sample of parents. This study also utilized longitudinal data to determine whether self-reported dispositional empathy predicted EMPAT scores.

### Participants

Participants were 212 parents (mothers *n* = 119; fathers *n* = 93) from Time 4 of a prospective, longitudinal study, the “Following First Families” (Triple-F) study. Participants initially invited for the Triple-F Study were a sample of first-time mothers and their male partners, recruited in their third trimester of pregnancy from community health centers and ob/gyn clinics, with over half involving families with one or more sociodemographic risks (i.e., ≤150% of the federal poverty line, receipt of federal assistance, ≤ high school education, single parenthood, ≤ age 18). The larger three-wave study began with 203 primiparous mothers and 151 of their partners (Time 1). To remain eligible to continue in the study, mothers needed to have custody of the focal child. Of the 201 eligible families, parents were reassessed for Time 2 (*n* = 186 mothers, 146 fathers) when children were 6 months old (± 2 weeks) and for Time 3 (*n* = 180 mothers, 144 fathers) when children were 18 months old (± 3 weeks). Families were re-invited for this study for Time 4 when children were between 4 years and 4 years, 6 months.

At Time 4, 50.9% of the sample identified their race as White and 48.1% identified as Black; the remaining 1% identified as Asian (0.5%) or Native American/Native Alaskan (0.5%); of the full sample, 7.5% identified as biracial and 5% identified as Hispanic or Latinx. Approximately half of mothers in the current sample (*M*_age_ = 30.44, *SD*_age_ = 5.80) reported a combined household income less than $50,000 annually, with 32.8% receiving public assistance. Mothers reported educational level: 23.5% had a high school diploma or less, 25.2% completed some college, 22.7% completed a four-year college degree, and 28.6% had completed more than a four-year degree. Fathers in the current sample (*M*_age_ = 32.65, *SD*_age_ = 6.75) reported their educational attainment: 24.8% reported a high school diploma or less, 30.1% had completed some college, 20.4% had completed a four-year college degree, and 24.8% had completed more than a four-year degree.

Participants in the current sample (Time 4) did not differ significantly from participants not retained from the three previous time points on sociodemographic variables such as age, socio-economic status, employment status, or minority status. However, participants who unmarried at Time 1 [χ2 (1, 191) = 4.24, *p* < .05] and at Time 3 [χ2 (1, 189) = 9.50, *p* < .01] were more likely to be missing from the sample by Time 4.

### Measures

#### EMPAT-EA

Eight audio clips for the EMPAT-EA were used in Study 2 (Boy/Girl Happy, Mad, Sad, Scared). Participants were asked to imagine their child expressing the target emotions when listening to the audio stimuli. Participants in this study used the 5-point Likert scale to rate how hearing the child in each clip made them feel (EMPAT-EA Self) for emotions in the following order: Happy, Sad, Mad, Scared, Irritated, Worried, Cheerful, and Blue. To identify child emotion (EMPAT-EA Child), they rated each emotion audio clip in the following order: Happy, Sad, Mad, Scared, Angry, Afraid, Cheerful, Irritated, Worried, and Blue.

#### EMPAT-ES

The Child version was used in this study, with two scripts per emotion (Happy, Mad, Sad, Scared) balanced by child gender, with the same emotion response options as the EMPAT-EA Child audio version. Participants were asked to imagine their child expressing the target emotions when reading the script stimuli.

#### Convergent validity measures

*Interpersonal reactivity index*. The IRI [44] was again used in the current study as a self-report measure of dispositional empathy. The IRI was administered in the Triple-F study at every time point, permitting an examination of how earlier empathic responding related to the EMPAT tasks. The IRI demonstrated good reliability across time points (α = .80 to .86).

*Parental empathy measure*. The PEM [20] is a 25-item self-report measure of parents’ empathy directed toward their own child. The measure includes two subscales—Cognitive and Affective empathy. Parents respond to items on a 5-point Likert scale from 1 (*Not at all true*) to 5 (*Very true*) (e.g., “When my child is angry, I feel angry on his/her behalf”). Items are summed to create a total score and subscale scores, with higher scores on each reflecting greater cognitive or affective empathy directed toward their own child. The test authors report the PEM correlates with coded parental interviews of empathy toward their child. The PEM demonstrated good internal consistency in the current sample (α = .81).

*Parental empathy quotient-short*. The EQS [43] used in Study 1 is a measure of dispositional empathy. The Parental Empathy Quotient-Short (P-EQS) modified 19 EQS items, rephrased to reflect parents’ perspective-taking ability and responsiveness directed toward their own child [47]. The P-EQS significantly relates to self-reported dispositional empathy, as measured by the IRI, as well as parental empathy (particularly cognitive empathy), as measured by the PEM, evidencing validity [47]. In the P-EQS, participants indicate their agreement on a 4-point Likert scale to items suggestive of child-directed empathy (e.g., “I am good at predicting how my child will feel”). Higher scores are indicative of greater parental empathy. The measure demonstrated good internal consistency (α = .83) in the current sample.

#### Concurrent validity measures

*Parental authority questionnaire*. The PAQ [48] is a measure of parenting styles. Although three parenting styles can be assessed, Authoritative parenting was the focus in the current study as most theoretically relevant to positive parenting (e.g., “I set firm guidelines for my children, but I am understanding when they disagree with me”). With 10 items on a 5-point Likert scale, higher scores on the subscale indicate greater levels of Authoritative parenting. The test authors report good reliability and concurrent validity. The PAQ Authoritative subscale demonstrated good internal consistency in the current sample (α = .84).

*Parenting young children scale*. The PARYC [49] is a 21-item measure designed to assess parenting behaviors for parents of children below age 6. Consisting of three subscales, Supporting Positive Behavior was selected for this study as most relevant to positive parenting. Items on this subscale (e.g., “Notice and praise your child’s good behavior?”) are rated for how often they engaged in each behavior in the past month on a 7-point Likert scale from 1 (*Not at all*) to 7 (*Most of the time*). The PARYC has demonstrated concurrent validity with other parenting measures, with fewer maladaptive parenting strategies and child problem behaviors. The PARYC Supporting Positive Behavior subscale demonstrated good internal consistency in the current study (α = .91).

*Response analog to child compliance task*. The ReACCT [50] is an analog task designed to assess harsh parenting which presents a realistic parent-child scenario in which the parent is running late for preschool. A series of sequential scenes are presented where the parent is portrayed as providing an instruction to the child in order to get ready to leave for preschool and the child responds with either compliance or non-compliance. A total of 20 steps are scored because the parent can remain “stuck” in a scene if the child remains noncompliant. At each step, parents select from 16 possible adaptive or maladaptive responses to the child’s behavior, which receive positive or negative weights, respectively. Throughout the task, participants hear and see a ticking clock to elicit urgency, and each time the parent appears to obtain quick compliance, the parent receives a game bonus of 50 cents. ReACCT can be scored for parents’ responses to children’s Noncompliance (12 items) and Compliance (8 items), with higher scores on both subscales indicating harsher responses. For the current study, only parents’ Compliance subscale scores were used to estimate parents’ positive parenting responses to compliance. Across several samples of varying risk, higher ReACCT scores are associated with measures of child abuse risk and abusive discipline tactics [50]. The measure demonstrated good internal consistency in the current sample (α = .85).

### Procedures

Parents completed measures in a 2-hour session, and all measures were presented with Inquisit 4.0 software via laptop computers with headphones. After providing informed consent, the EMPAT-EA Self was administered first in the protocol, followed by a series of measures that included dispositional and parental empathy and positive parenting; the EMPAT-EA Child and EMPAT-ES Child were presented at the end of the full protocol. All study procedures for each wave of this longitudinal study, including Time 4, were approved by the University of Alabama at Birmingham Institutional Review Board (IRB-130626002).

### Analytic plan

Comparable to Study 1, the emotion subscale factors for each version of the EMPAT in the parent sample were again determined through exploratory factor analysis, seeking to identify the most parsimonious solution, using scale reliability and ITCs to confirm these compositions, adopting the same set of minimum criteria for each step. For interpretation of concurrent and convergent validity, of primary interest are the total scores for each version.

### Results

#### Confirmation of stimuli accuracy

Accuracy of the stimuli was confirmed using ratings from the EMPAT child versions. For EMPAT-EA audio stimuli, 92.4% and 91.9% of respondents rated the Boy and Girl Happy audios, respectively, as “extremely” or “quite a bit” happy. The EMPAT-EA Boy and Girl Mad audios were rated as “extremely” or “quite a bit” mad by 83.3% and 89.1% of parents, respectively. EMPAT-EA Sad Boy and Girl audio stimuli were correctly identified by 74.8% and 84.7% of respondents, respectively. The EMPAT-EA Scared audios were correctly identified as scared by 70.0% and 90.0% of respondents for the Boy and Girl audio, respectively. For the EMPAT-ES script stimuli, 87.2% and 85.2% of parents rated the two Happy scripts as “extremely” or “quite a bit” happy. The two EMPAT-ES Mad scripts were rated as “extremely” or “quite a bit” Mad by 83.3% and 75.8% of parents. The EMPAT-ES Sad scripts were correctly identified by 82.8% and 80.0% of parents. The two EMPAT-ES Scared scripts were rated as “extremely” or “quite a bit” scared by 80.9% and 85.3% of respondents.

#### Factor analysis

See Table 3 for the final items included in each subscale. For the EMPAT-EA Child audio stimuli, the Happy subscale included 20 items (10 emotions) which explained 82.1% of the variance. The Mad subscale consisted of 10 items (5 emotions) explaining 54.5% of the variance. The Sad subscale was comprised of 8 items (4 emotions) explaining 61.0% of the variance. The Scared subscale consisted of 10 items (5 emotions) that explained 59.9% of the variance.

**Table 3:** Study 2: Emotion subscale scoring approach based on exploratory factor analyses (with EFA factor loadings for boy/girl versions of the stimuli).

	Study 2: Emotion Subscale Components
**EMPAT-EA Child**	
Happy	Happy (.63, .66), Cheerful (.75, .76), Sad* (.69, .89), Mad* (1.0, .91), Scared* (1.0, .96), Angry* (.89, 1.0), Afraid* (.92, 1.0), Irritated* (.89, .91), Worried* (.73, .92), Blue* (.96, .91)
Mad	Mad (.66, .76), Angry (.72, .78), Irritated (.55, .63), Sad (.69, .77), Blue (.84, .82)
Sad	Sad (.78, .67), Blue (.84, .77), *Happy (.77, .79), *Cheerful (.75, .81)
Scared	Scared (.87, .51), Afraid (.86, .58), Worried (.81, .56), Happy* (.78, .75), Cheerful* (.90, .81)
**EMPAT-EA Self**	
Happy	Happy (.86, .84), Cheerful (.86, .85), Mad* (.94, .94), Sad* (.93, .97), Scared* (.94, .92), Irritated* (.95, .85), Worried* (.97, .87), Blue* (.76, .92)
Mad	Sad (.91, .82), Worried (.92, .86), Blue (.72, .80), Scared (.81, .96)
Sad	Sad (.80, .80), Worried (.77, .93), Blue (.82, .61), Scared (.80, .72)
Scared	Sad (.77, .90), Worried (.97, .60), Blue (.58, .89), Scared (.86, .74)
**EMPAT-ES Child**	
Happy	Happy (-.66, -.52), Cheerful (-.63, -.46), Sad* (.84, .86), Mad* (.92, .88), Scared* (.90, .75), Angry* (.87, .90), Afraid* (.89, .76), Irritated* (.85, .86), Worried* (.85, .82), Blue* (.85, .90)
Mad	Mad (.59, .87), Angry (.57, .82), Irritated (.73, .83), Cheerful* (.87, .90), Happy* (.90, .81)
Sad	Sad (.57, .56), Blue (.75, .77), Worried (.67, .86), Cheerful* (.86, .81), Happy* (.73, .75)
Scared	Scared (.74, .81), Afraid (.77, .81), Worried (.81, .83), Happy* (.56, .59), Mad* (.77, .77), Angry* (.79, .79), Cheerful* (.59, .46), Irritated* (.73, .73)

*Note*. Emotion words on the right indicate responses that met minimum criteria in the exploratory factor analysis for the respective emotion factor and were then included in the corresponding EMPAT emotion subscale score.

*indicates the response was reverse scored.

For the EMPAT-EA Self audio stimuli, the Happy subscale included 16 items (8 emotions) that explained 81.5% of the variance. The Mad subscale consisted of 8 items (4 emotions) that explained 74.7% of the variance. The Sad subscale was comprised of 8 items (4 emotions) that explained 63.6% of the variance. The Scared factor was comprised of 8 items (4 emotions) that accounted for 67.3% of the variance.

For the EMPAT-ES Child script stimuli, the Happy subscale included 20 items (10 emotions) accounting for 76.9% of the variance. The Mad subscale consisted of 10 items (5 emotions) that explained 66.9% of the variance. The Sad subscale included 10 items (5 emotions) that explained 59.2% of the variance. The Scared subscale consisted of 16 items (8 emotions) that accounted for 58.9% of the variance.

#### Reliability

See Table 4 for internal consistency coefficients. For EMPAT-EA Child audio stimuli, the Total score demonstrated excellent reliability and all emotion subscales (i.e., Happy, Mad, Sad, Scared) met the minimum alpha of at least .70. Examination of the responses included in each emotion subscale indicated that Happy (.31-.84), Mad (.36-.59), and Scared (.37-.67) all met the minimum criteria for item-total correlations of at least .30. However, the range of item total correlations for the EMPAT-EA Child Sad subscale fell below this cutoff (.23-.57). Because removal of that item did not improve alpha, the item was retained to preserve its comparable item for the other gender. For EMPAT-EA Self audio stimuli, the Total score also demonstrated excellent internal consistency and all emotion subscales exceeded all criteria. EMPAT-EA Self Happy (.38-.77), Mad (.65-.73), Sad (.46-.64), and Scared (.54-.72) subscales all demonstrated acceptable item total correlations. For the EMPAT-ES Child script stimuli, the Total score demonstrated excellent reliability and all emotion subscales exceeded the minimum criteria set for internal consistency and ITCs. For EMPAT-ES emotion subscale, ITCs were as follows: Happy (.47-.88), Mad (.40-.72), Sad (.35-.70), and Scared (.40-.66).

**Table 4:** Study 2: Means, standard deviations, reliability, and cross-sectional validity correlations for EMPAT tasks.

	*M (SD)*	α	PEM_T_	PEM_C_	PEM_A_	P-EQS	IRI_EC_	IRI_PT_	PAQ	PARYC_S_	ReACCT
***Audio-Self***											
Happy	4.80 (0.34)	.87	.27***	.29***	.19*	.18**	.19**	.16*	.17*	.13	-.12
Mad	2.47 (1.08)	.90	.22***	.15*	.25***	.15*	.07	.01	.08	.04	-.09
Sad	2.48 (0.86)	.84	.15*	.09	.19**	.09	.12	.10	.10	.14*	-.19**
Scared	2.88 (0.93)	.86	.18**	.12	.20**	.13	.12	.07	.11	.16*	-.12
Total	12.63 (2.58)	.92	.24***	.17*	.27***	.16*	.14*	.09	.13	.14	-.16*
***Audio-Child***											
Happy	4.83 (0.44)	.94	.17*	.20**	.10	.19**	.25***	.20**	.29***	.22**	-.35***
Mad	3.61 (0.77)	.78	.29***	.24***	.28***	.28***	.24***	.28***	.20**	.24***	-.32***
Sad	4.35 (0.59)	.70	.23***	.24**	.16*	.23**	.31***	.31***	.32***	.27***	-.41***
Scared	4.40 (0.58)	.80	.30***	.28***	.26***	.25***	.29***	.22***	.32***	.29***	-.37***
Total	17.19 (1.87)	.92	.33***	.31***	.27***	.31***	.35***	.33***	.35***	.33***	-.46***
***Script-Child***											
Happy	4.69 (0.62)	.96	.22***	.23***	.16*	.27***	.40***	.32***	.36***	.28***	-.31***
Mad	4.36 (0.65)	.87	.24***	.23***	.20**	.21**	.35***	.26***	.34***	.26***	-.40***
Sad	4.24 (0.69)	.84	.33***	.30***	.28***	.26***	.41***	.27***	.39***	.32***	-.41***
Scared	4.29 (0.64)	.86	.23***	.24***	.17	.17	.43***	.30***	.40***	.28***	-.42***
Total	17.57 (2.29)	.96	.29***	.28***	.23**	.26***	.45***	.32***	.42***	.32***	-.44***
***M* (*SD*)**			109.68 (9.21)	62.17 (5.49)	47.50 (4.88)	63.31 (7.23)	28.59 (4.94)	27.48 (5.14)	41.63 (6.54)	43.63 (6.30)	-10.06 (9.83)

*Note*. PAQ = PEM_T_ = Parental Empathy Measure-Total score; PEM_C_ = Cognitive scale; PEM_A_ = Affective scale; P-EQS = Parental Empathy Quotient-Short; IRI_EC_ = Interpersonal Reactivity Index- Empathic Concern scale; IRI_PT_ = Perspective Taking Scale; Parental Authority Questionnaire, Authoritative; PARYC_S_ = Parenting Young Children-Supporting Positive Behavior scale; ReACCT = Response Analog to Child Compliance Task, Compliance Scale.

*p ≤ .05,

**p ≤ .01,

***p ≤ .001.

#### Validity

*Concurrent validity*. The EMPAT-EA Self Total score and all four emotion subscale scores were significantly related to affective (i.e., empathic concern) aspects of empathy as assessed by the Parental Empathy Measure (see Table 4). Only the EMPAT-EA Self Total score, Happy, and Mad subscales were associated with cognitive (i.e., emotion recognition, perspective taking) aspects of empathy on the PEM (Table 4). The Total score and Happy and Mad subscale scores of the EMPAT-EA Self were also associated with the other self-report measure of parental empathy (P-EQS). Only the EMPAT-EA Self Happy subscale score was related to the dispositional measure of empathy, the IRI, concurrently (Table 4) and longitudinally with empathic concern (see Table 5). However, this Happy subscale only became significantly associated with the dispositional measure of empathy at Time 2, after parents transitioned into parenthood (see Table 5). The total EMPAT-EA Self score was unrelated to IRI scores across time.

**Table 5:** Means, standard deviations, and longitudinal validity correlation coefficients for the EMPAT tasks for parents in Study 2.

	IRI_EC (T1)_	IRI_PT (T1)_	IRI_EC (T2)_	IRI_PT (T2)_	IRI_EC (T3)_	IRI_PT (T3)_
*EMPAT-EA Self*	*r*	*r*	*r*	*r*	*r*	*r*
Happy	.10	-.01	.16*	.02	.17*	.02
Mad	-.01	-.04	.01	.01	-.04	-.00
Sad	.04	-.03	.03	.05	.02	.02
Scared	.02	-.04	.02	-.07	.00	-.02
Total	.03	-.04	.04	-.01	.01	.00
***EMPAT-EA Child***						
Happy	.19**	.05	.18*	.08	.20**	.04
Mad	.09	.07	.12	.10	.14	.15*
Sad	.24***	.14*	.24***	.16*	.21**	.13
Scared	.15*	.02	.22**	.10	.17*	.07
Total	.20**	.10	.23**	.14	.22**	.14
***EMPAT-ES Child***						
Happy	.28***	.12	.29***	.13	.28***	.18*
Mad	.31***	.09	.37***	.20**	.33***	.16*
Sad	.33***	.08	.35***	.12	.36***	.17*
Scared	.27***	.11	.33***	.15*	.36***	.17*
Total	.34***	.11	.38***	.17*	.37***	.20**
*M* (*SD*)	28.18 (4.57)	26.41 (4.69)	27.75 (4.81)	26.77 (5.00)	27.60 (4.61)	26.71 (4.65)

*Note*. IRI = Interpersonal Reactivity Index; T1 = Time 1 prenatal assessment, T2 = Time 2 children 6 mo. old, T3 = Time 3 children 18 mo. old.

*p ≤ .05,

**p ≤ .01,

***p ≤ .001.

The Total score and all emotion subscales of the EMPAT-EA Child audio version (except for Happy) were significantly related with scores on the self-reported parental empathy measure (PEM Total, PEM Cognitive, and PEM Affective; see Table 4). All EMPAT-EA Child scores were also significantly related to the other parental empathy measure (P-EQS; see Table 4). The EMPAT-EA Child Total (as well as all four emotion subscales) score was also significantly associated with self-reported affective empathy (IRI empathic concern) and cognitive empathy (IRI perspective taking) concurrently (i.e., at Time 4; Table 4), and most of the emotion subscales were longitudinally related to empathic concern on the IRI across all four time points (spanning four years; Table 5). However, the significant relationship between EMPAT-EA Child Mad and dispositional empathy on the IRI was not apparent until Time 3 (see Table 5) and was associated with cognitive (IRI perspective taking), rather than affective empathy. Interestingly, the EMPAT-EA Child emotion subscale scores demonstrated more longitudinal associations with aspects of affective empathy (i.e., empathic concern) than cognitive empathy, counter to what was expected of the Child scales.

For the script version, the EMPAT-ES Child Total score and all emotion subscales were significantly related to cognitive aspects of parental empathy (PEM; Table 4). The EMPAT-ES Child Total score (but not Scared subscale scores) was associated with affective aspects of parental empathy on the PEM (Table 4). Similarly, all emotion subscale scores except for the Scared score on the EMPAT-ES Child were significantly related to the other measure of parental empathy (P-EQS; Table 4). By contrast, the EMPAT-ES Child demonstrated significant associations with all aspects of the self-reported dispositional empathy measure (IRI). Specifically, all four emotion subscales scores and the Total score of the EMPAT-ES Child were significantly related to both cognitive and affective aspects of dispositional empathy (IRI; see Table 4). These relationships were also apparent longitudinally at Time 3; note the Total score and all emotion subscale scores of the EMPAT-ES Child were significantly related to affective aspects of dispositional empathy as early as Time 1 (i.e., across four years; Table 5).

*Concurrent validity*. See Table 4 for concurrent validity correlations. For the EMPAT-EA Self version, only the Happy subscale of the audio version was significantly related to concurrent, self-reported Authoritative parenting (not the total and other three emotion subscales). Self-reported supportive parenting practices (PARYC-Supporting Positive Behavior) were significantly related to EMPAT-EA Self Total score and all emotion subscale scores except for Mad. Similar to patterns observed with the PARYC, all emotion subscale scores except for Mad were significantly related to parents’ adaptive responses to children’s compliance (ReACCT Compliance score).

For the EMPAT-EA Child audio analog task, all four target emotion subscales and the Total score were significantly related to concurrent, self-reported authoritative parenting (PAQ), parenting that would support children’s positive behavior (PARYC), and parents’ adaptive reactions to children’s compliance (ReACCT Compliance). Similarly, all four emotion subscales and the Total score of the EMPAT-ES Child script analog were significantly related to concurrent, self-reported authoritative parenting (PAQ Authoritative), parenting supportive of children’s behavior (PARYC Supporting Positive Behavior scale), and parents’ adaptive responses to children’s compliance (ReACCT Compliance scale).

### Discussion

Results of this second study provide initial support for the reliability and validity of novel analog measures of both EMPAT versions, the EMPAT-EA (audio) and EMPAT-ES (script), in a parent sample. Both the EMPAT audio and script stimuli were accurately identified by most parents. Further, the subscale and total scores for the EMPAT-EA Self and Child audio versions, as well as the EMPAT-ES Child script version, demonstrated acceptable internal consistency.

The EMPAT-EA Self using audio stimuli exhibited convergent validity with both cognitive and affective subscales of a self-report measure of parental empathy (PEM); however, fewer associations between the EMPAT-EA Self with another self-report measure of parental empathy (P-EQS) and dispositional empathy (IRI) were identified. The greater number of significant associations between parental empathy, as opposed to dispositional empathy, and parents’ own reaction to children’s emotions is a possible indicator that parental empathy may be more related to parents’ own emotional responses than their children’s. Specifically, identifying children’s emotions may be a more general empathic skill whereas appropriately responding to your child’s emotions may reflect the child-directed nature of parental empathy. Further, although parents’ responses to how hearing a child’s emotion made them feel was expected to primarily evoke affective empathy, the EMPAT-EA Self subscales evidenced relations with both affective and cognitive empathy. Similarly, the EMPAT-EA Child scores for audio stimuli exhibited consistent significant relations across measures, with evidence of associations with self-reported parental (PEM, P-EQS) and dispositional affective and cognitive empathy (IRI). Parents’ ability to identify children’s sad emotions (i.e., EMPAT-EA Child Sad score) was related to empathy even prior to and throughout early parenthood, which may implicate parents’ ability to recognize children’s distress as a key factor in expressing appropriate parental empathy [51].

Similar to the Child scores using audio stimuli, the EMPAT-ES Child scores using evocative scripts were related to self-reported parental cognitive empathy (PEM, P-EQS) and dispositional empathy. The Mad and Scared subscales of the EMPAT-ES Child were the most consistent correlate of parents’ empathy even prenatally, again indicating that parents’ ability to recognize children’s distress emotions may be particularly important features of parental empathy [51]. Previous research on how individuals appraise and regulate emotion also suggests that outcomes may be influenced by the interface between the valence of the emotion (i.e., positive, negative) and the type of evocative stimuli (i.e., verbal, non-verbal) [52]. Interestingly, for the EMPAT-ES Child script stimuli, the Scared subscale score was not significantly related to a measure of parental empathy (P-EQS), including the affective subscale of one of the parental empathy measures (PEM), which may indicate a parent’s ability to identify fear may be lacking in the self-report measures because this subscale was related to the other measures of dispositional empathy and positive parenting. Given the strong performance of the EMPAT-ES Total score, these findings underscore the value of including multiple child emotions.

The expectation that parents’ reactions to hearing children’s emotions (i.e., EMPAT-EA Self) would be positively related to authoritative parenting, supportive parenting behavior, and adaptive reactions to child compliance was only partially supported. Specifically, only parents’ reactions to hearing children’s happiness were related to self-reported Authoritative parenting. However, parents’ reactions to children’s Happy, Sad, and Scared emotions were related to supportive parenting and adaptive reactions to compliance. Interestingly, parents’ reactions to children’s Mad emotions were unrelated to any of these markers of positive parenting. Past research evidencing the distinct nature of positive parenting (i.e., rather than an inverse) from harsh parenting [11] may help explain this finding; parents’ adaptive reactions to children’s happiness or sadness may better predict positive parenting than less harsh parenting. However, parents’ ability to accurately identify children’s emotions, as indicated by EMPAT-EA Child and EMPAT-ES Child Total scores, was related to all positive parenting indices. Previous research on parental sensitivity and responsiveness indicates the necessity of accurate emotion recognition for positive parenting [22], which may account for these differences in associations between the parenting measures and the EMPAT Self versus Child versions. Parents’ cognitive parental empathy, as measured in the Child versions, may be a potentially more salient predictor of positive parenting than affective empathy.

## General discussion

In the current investigation, the EMPAT was introduced as a set of new measurement tools to objectively and reliably assess the ability to accurately identify and appropriately respond to children’s emotions in order to address the need for implicit, easily administered measures of parental empathy [11,20,27]. Two versions of the task were created which used different stimuli: the EMPAT-EA utilized audio clips of young children expressing four target emotions (Happy, Sad, Mad, Scared) and the EMPAT-ES used evocative scripts detailing scenarios likely to elicit each of the four target emotions in a young child. In both versions, participants were asked to indicate how the emotion stimuli made them feel (i.e., Self responses) and how the child in the emotion stimuli felt (i.e., Child responses) in order to elicit affective and cognitive aspects of empathy, respectively. Because of the ambiguity of what is required in responding to the stimuli, the tasks would minimize socially desirable responding. Additionally, this initial validation of the current analog measures was intended to reduce the need for equipment or lengthy parent interviews that are laborious for participants, interviewers, and data coders, alike. The analog tasks were first piloted with a sample of university students (Study 1) and then with a parent sample involved in a larger longitudinal study (Study 2). Results of both studies provide preliminary support for the reliability and validity of the EMPAT tasks as implicit measures of parental empathy.

In Study 1, both versions of the EMPAT demonstrated concurrent validity with self-report measures of dispositional empathy in an undergraduate student sample. In particular, the EMPAT exhibited significant correlations with self-reported dispositional empathic concern, suggesting the utility of the analog tasks to measure affective empathy. Although the Self versions were expected to evoke affective empathy and the Child versions were expected to evoke cognitive empathy, all versions were largely related to dispositional affective empathy. Interestingly, only the EMPAT-ES Child scores demonstrated relationships with cognitive empathy in the undergraduate sample. Because EMPAT-ES Self exhibited relative problems in assessing empathy due to concerns regarding their reactions to mad scripts (a critical emotion relevant for parents), this version was omitted in the subsequent study of parents.

In Study 2, improved accuracy in identifying target emotions was observed across all versions of the EMPAT suggesting that modifications to emotion stimuli were largely successful. Contrary to results of the pilot study emphasizing affective empathy, the EMPAT appeared more successful in tapping cognitive empathy in the parent sample. The Child versions of the analog task, which were expected to capture parents’ cognitive empathy (emotion recognition), demonstrated more consistent associations with self-report measures of parental empathy and positive parenting. Such distinct patterns between study samples may indicate that cognitive empathy, and accurate emotion identification specifically, may be more essential for successful parenting. Previous research demonstrating the strength of the relationship between parents’ cognitions about children and their resultant parenting behavior [53] may implicate that parents’ cognitive attributions about children may drive parents’ cognitive empathy. Still others have pointed to the necessity of parents’ affective empathy abilities as both a factor in more sensitive parenting [22] and in lower child abuse risk [13], which argues for the importance of both aspects of empathy within the context of parenting. The possibility remains that aspects of cognitive and affective empathy may not be so easily differentiated; rather, these components may simply contribute to parallel empathic processes.

The difficulty in measuring parents’ affective empathy in Study 2 may be due, in part, to the omission of EMPAT-ES Self given that EMPAT-EA Self demonstrated more consistent associations with self-reported parental affective empathy. Continued development of the EMPAT versions is needed to enhance measurement of parents’ affective empathy. For example, further development may involve presenting emotion script scenarios that attempt to match the stimuli more closely to their particular child (e.g., by child gender, by child age in the scripts) rather than simply having to imagine that the figurative child is their own. Previous research using evocative scripts has also implicated the utility of presenting audio of pre-recorded emotion scripts in order to evoke a target emotion [54] rather than participants simply reading an emotion script, which may have been more effective for parents despite the problems with EMPAT-ES Self in the undergraduate sample. Relative to the Child scores, measurement of affective parental empathy was more effective with the EMPAT-EA Self, with audio Self scores largely related to the explicit PEM [20]; but its relationships with positive parenting were variable, thereby limiting its utility.

Another explanation of the EMPAT assessments’ performance in the current sample may be that the tasks measured specific skills within the broader categories of affective and cognitive empathy, which were limited by the available choices for validity measures. The EMPAT Child tasks appeared to clearly assess parents’ emotion identification abilities (a skill within cognitive empathy) rather than solely perspective taking skills wherein associations with cognitive empathy skills remained consistent. However, despite expectations that the EMPAT Self tasks would assess affective empathy, the ability of the EMPAT Self tasks to uniquely assess affective empathy was not observed. These results may be explained by previous research describing a distinction within affective empathy skills that are self-focused versus other-focused [see 19]. The EMPAT Self tasks more obviously aim to assess self-focused empathy skills (i.e., emotional resonance) yet the current study utilized an other-focused measure of affective empathy (IRI Empathic Concern). This distinction may explain the surprising results that suggested affective empathy was difficult to distinguish between the different versions of the EMPAT tasks.

Both studies provide initial evidence for the efficacy of the novel EMPAT analogs in measuring parental empathy. Although considerable research about the theoretical underpinnings of cognitive and affective empathy exists [2–6], the analog measures in the current investigation did not advance the speculation that empathy involves discriminable cognitive and affective processes. Rather, the EMPAT assessments suggest the relative importance of parents’ ability to recognize children’s emotions. An overarching goal of the current research project was to develop an analog measure of empathy specific to parenting. The ability of the analog tasks to measure parents’ child-directed empathy and the analogs’ relationships with multiple indices of positive parenting suggest the value of creating and using parent-specific measures of empathy in the context of studying parenting beliefs and behaviors.

### Limitations and future research directions

Although efforts in developing and validating the novel EMPAT-EA and EMPAT-ES tasks were systematic—as demonstrated by the multi-study process by which the analog tasks were created, pilot tested, and improved—our results are preliminary and some limitations remain. Changes to the EMPAT protocol between the two studies may have compromised the conclusions from the current research. Although exclusion of the EMPAT-ES Self version seemed necessary based on results of Study 1 (parents’ reactions to children’s anger were deemed critical), its omission from Study 2 limited the ability to determine how well each version of the tasks (i.e., Self versus Child) were assessing different aspects of parent-specific empathy. Further, because EMPAT Self versions were intended to elicit aspects of affective empathy, inclusion of both EMPAT Self analogs may have provided more insights into how parents’ affective empathy is related to other facets of parenting, distinct from cognitive empathy. Nearly universal associations between EMPAT scores and self-reported cognitive empathy in the parent sample suggest that cognitive and affective empathy may not be distinguished using the current task stimuli. Future work should consider how well parents’ reactions to unique child emotions (e.g., happiness, anger, sadness, fear) reflect parent empathic abilities (i.e., cognitive, affective) and relate to other measures of parenting. Relatedly, because reactions to stimuli depicting child “mad” emotions did not perform as expected across studies, future work on the EMPAT should consider how adaptive reactions to children’s anger may be better represented by parents’ lower levels of personal distress, as not responding to child frustration with maladaptive reactions would be particularly informative. Finally, additional indices of parent factors that may contribute to, or predict, parental empathy, such as parents’ child behavior attributions, emotion regulation, or frustration tolerance, were not included in the current study but may provide useful insights into the relationship between empathy and parenting. Additional validation of the EMPAT tasks will rely on further study in relation to additional indices of concurrent validity as well as discriminant validity in order to demonstrate both research and clinical utility.

Pilot testing the analog tasks with a sample of university students in Study 1 was cost effective and did allow for initial modifications prior to testing with the target parent sample; however, some unexpected results from Study 1 may be attributable to the study sample not representing the intended population (i.e., parents) for the analog tasks. Although some significant patterns between the EMPAT scales and self-reported empathy emerged in Study 1, the possibility remains that pilot testing with an initial parent sample may have resulted in fewer or different modifications to the analog tasks. Additionally, responses from the undergraduate sample in Study 1 may have been influenced by participants’ previous babysitting or caregiving experiences; however, information about these experiences was not collected in the current study. Future research may consider how early caregiving experiences may shape future parental empathy. Parents in the current sample had children below 5 years, which may limit the ability to fully understand how parental empathy unfolds as children age. Specifically, parental empathy may be partially dependent on children’s development, maturity, and communication abilities.

In the future, more theoretical work is needed in order to determine additional ways in which parental and dispositional empathy differ. For instance, although the current parental empathy analog measures were created within the framework used to delineate cognitive and affective aspects of dispositional empathy, parental empathy may be better understood outside of the cognitive-affective distinctions influenced by the parenting-specific context, such as the nature of the attachment relationship and beliefs about parenting [11]. Future research would benefit from considering how parental empathy may be embedded in other facets of parenting (e.g., child-directed attributions and emotion regulation). This future goal may also be methodologically strengthened with inclusion of direct observation of parent-child interactions to explore the validity of the EMPAT-E as a predictor of actual parenting behavior. Although direct examination of measurement invariance by gender was not possible in the current study due to limited power, examination of how gender of the parent as well as gender of the participants’ children influence parental empathic responding on the EMPAT-E is a critical future direction. Further, although the parent sample in Study 2 evidenced racial diversity, differences in parental empathy and positive parenting were not considered with respect to ethnic or racial identification in the current study. Future work on the EMPAT tasks should also examine measurement invariance by parents’ racial and ethnic background using a larger sample size to better understand the utility of the tasks for a diverse sample of parents. Finally, replication in other at-risk parent samples to further assess validity as well as adaptation of the analog tasks for childcare providers and schoolteachers is a necessary future direction in order to understand the efficacy and clinical utility of the EMPAT tasks.

### Conclusions

The significant impact that empathic abilities bear on parenting has long been demonstrated in psychological research, yet such research largely relies on measures of dispositional empathy to characterize processes specific to the parent-child relationship [12,15,27,28]. The present work indicates that empathy measures specific to parents may be useful in understanding indices of positive parenting behavior. The EMPAT tasks are a promising novel approach to assessing parents’ ability to understand and empathically respond to children’s emotions in a relatively brief manner that does not require lengthy protocols or significant coding efforts. Because measurement of parents’ cognitive empathy was more salient in the current research findings, more work on the implicit assessment of parents’ affective empathy in particular is still warranted.

## References

[1] Batson CD. These things called empathy: Eight related but distinct phenomena. In: Decety J & Ickes W, editors. The social neuroscience of empathy. Cambridge (MA): MIT Press; 2009. p. 3–15.

[2] Decety J, Jackson PL. The functional architecture of human empathy. Behav Cogn Neurosci Rev. 2004; 3(2): 71–100. doi: 10.1177/1534582304267187 15537986

[3] Davis MH. A multidimensional approach to individual differences in empathy. J Pers Soc Psychol. 1980; 10 (85): 2–19. Available from: https://www.uv.es/~friasnav/Davis_1980.pdf.

[4] Davis MH. Empathy: A social psychological approach. Boulder (CO): Westview Press; 1996.

[5] Eisenberg N, Eggum ND. Empathic responding: Sympathy and personal distress. In: Decety J, Ickes W, editors. The social neuroscience of empathy. Cambridge (MA): MIT Press; 2009. p. 71–83.

[6] Shamay-Tsoory SG, Aharon-Peretz J, Perry D. Two systems for empathy: a double dissociation between emotional and cognitive empathy in inferior frontal gyrus versus ventromedial prefrontal lesions. Brain. 2009; 132 (3): 617–627. doi: 10.1093/brain/awn279 18971202

[7] Batson CD, Ahmad N, Lishner DA, Tsang JA. Empathy and altruism. In: Snyder GR, Lopez SJ, editors. Handbook of positive psychology. London: Oxford University Press; 2002. p. 485–498.

[8] Jolliffe D, Farrington DP. Development and validation of the Basic Empathy Scale. J Adolesc. 2006; 29 (4): 589–611. doi: 10.1016/j.adolescence.2005.08.010 16198409

[9] Baron-Cohen S, Wheelright S. The empathy quotient: An investigation of adults with Asperger syndrome or high functioning autism, and normal sex differences. J Autism Dev Disord. 2004; 34 (2): 163–75. doi: 10.1023/b:jadd.0000022607.19833.00 15162935

[10] Knafo A, Zahn-Waxler C, Van Hulle C, Robinson JL, Rhee SH. The developmental origins of a disposition toward empathy: Genetic and environmental contributions. Emotion. 2008; 8(6): 737–52. doi: 10.1037/a0014179 19102585

[11] Kilpatrick KL. The Parental Empathy Measure: A new approach to assessing child maltreatment risk. Am J Orthopsychiatry. 2005; 75(4): 608–620. doi: 10.1037/0002-9432.75.4.608 16262518

[12] De Paul J, Perez-Albeniz A, Guibert M, Asla N, Ormaechea A. Dispositional empathy in neglectful mothers and mothers at high risk for child physical abuse. J Interpers Violence. 2008; 23(5): 670–84. doi: 10.1177/0886260507313532 18263864

[13] Perez-Albeniz A, De Paul J. Dispositional empathy in a high- and low-risk parents for child physical abuse. Child Abuse Negl. 2003; 27(7): 769–80. doi: 10.1016/s0145-2134(03)00111-x 14627078

[14] Rodriguez CM, Richardson MJ. Stress and anger as contextual factors and pre-existing cognitive schemas: Predicting parental child maltreatment risk. Child Maltreat. 2007; 12(4): 325–37. doi: 10.1177/1077559507305993 17954939

[15] Rodriguez CM, Tucker MC. Predicting maternal physical child abuse risk beyond distress and social support: Additive role of cognitive processes. J Child Fam Stud. 2015; 24:1780–90. doi: 10.1007/s10826-014-9981-9

[16] Wiehe VR. Empathy and narcissism in a sample of child abuse perpetrators and a comparison sample of foster parents. Child Abuse Negl. 2003; 27(5): 541–55. doi: 10.1016/s0145-2134(03)00034-6 12718962

[17] U.S. Department of Health & Human Services, Administration for Children and Families, Administration on Children, Youth, and Families. Child Maltreatment 2019. Children’s Bureau. 2021; https://www.acf.hhs.gov/sites/default/files/documents/cb/cm2019.pdf.

[18] Eisenberg N, Fabes RA, Schaller M, Carlo G, Miller PA. The relations of parental characteristics and practices to children’s vicarious emotional responding. Child Dev. 1991; 62: 1393–408. doi: 10.1111/j.1467-8624.1991.tb01613.x 1786723

[19] Ewing ESK, Herres J, Dilks KE, Rahim F, Trentacosta CJ. Understanding of emotions and empathy: Predictors of positive parenting with preschoolers in economically stressed families. J Child Fam Stud. 2019 Mar; 28: 1346–58. doi: 10.1007/s10826-018-01303-6

[20] Stern JA, Borelli JL, Smiley PA. Assessing parental empathy: A role for empathy in child attachment. Attach Hum Dev. 2015; 17(1): 1–22. doi: 10.1080/14616734.2014.969749 25373381

[21] Waters SF, Virmani EA, Thompson RA, Meyer S, Raikes HA, Jochem R. Emotion regulation and attachment: Unpacking two constructs and their association. J Psychopathol Behav Assess. 2010; 32(1): 37–47. doi: 10.1007/s10862-009-9163-z 20174446 PMC2821505

[22] Leerkes EM. Predictors of maternal sensitivity to infant distress. Parent Sci Pract. 2010; 10(3): 219–39. doi: 10.1080/15295190903290840 20824194 PMC2930822

[23] Fleckman JM, Taylor CA, Storer HL, Andrinopoulos K, Weil LEG, Rubin-Miller L, Theall KP. Breaking the mold: Socio-ecological factors to influence the development of non-harsh parenting strategies to reduce risk for child physical abuse. Child Youth Serv Rev. 2018; 94(2): 274–83. doi: 10.1016/j.childyouth.2018.10.019

[24] Borelli JL, Stern JA, Marvin MJ, Smiley PA, Pettit C, & Samudio M. Reflective functioning and empathy among mothers of school-aged children: Charting the space between. Emotion. 2020; 10.1037/emo0000747. 32191098

[25] Oppenheim D, Koren-Karie N, Sagi A. Mothers’ empathic understanding of their preschoolers’ internal experience: Relations with early attachment. Int. J. Behav. Dev. 2001; 25 (1), 16–26. http://www.tandf.co.uk/journals/pp/01650254.html.

[26] Strayer J, Roberts W. Children’s anger, emotional expressiveness, and empathy: Relations with parents’ empathy, emotional expressiveness, and parenting practices. Soc Dev. 2004; 13(2): 229–54. doi: 10.1111/j.1467-9507.2004.000265.x

[27] Francis KJ, Wolfe DA. Cognitive and emotional differences between abusive and non-abusive fathers. Child Abuse Negl. 2008; 32(12): 1127–37. doi: 10.1016/j.chiabu.2008.05.007 19036447

[28] Davidov M, Grusec JE. Untangling the links of parental responsiveness to distress and warmth to child outcomes. Child Dev. 2006; 77(1): 44–58. doi: 10.1111/j.1467-8624.2006.00855.x 16460524

[29] Bi S & Keller PS. Parental empathy, aggressive parenting, and child adjustment in a noncustodial high-risk sample. J. Interpers. Violence. 2019; 00 (0); 1–22. doi: 10.1177/0886260519870165 31422750

[30] DeGarmo DS, Reid JB, Knutson JF. Direct laboratory observations and analog measures in research definitions of child maltreatment. In: Feerick M, Knutson MF, Trickett FM, Flanzer SM, editors. Defining and classifying child abuse and neglect for research purposes. Baltimore (MD): Brookes. 2006. p. 297–332.

[31] Fazio RH, Olson MA. Implicit measures in social cognition research: Their meaning and use. Annu Rev Psychol. 2003; 54:297–327. doi: 10.1146/annurev.psych.54.101601.145225 12172003

[32] Baron-Cohen S, Wheelwright S, Hill J, Raste Y, Plumb I. The “Reading the Mind in the Eyes” Test Revised Version: A study with normal adults and adults with Asperger syndrome or high-functioning autism. J Child Psychol Psychiatry. 2001; 42(2): 241–51. doi: 10.1111/1469-7610.00715 11280420

[33] Kämpfe N, Penzhorn J, Schikora J, Dünzl J, Schneidenbach J. Empathy and social desirability: a comparison of delinquent and non-delinquent participants using direct and indirect measures. Psychol Crime Law. 2009; 15(1):1–17. doi: 10.1080/10683160802010640

[34] Asla N, de Paul J, Perez-Albeniz A. Emotion recognition in fathers and mothers at high-risk for child physical abuse. Child Abuse Negl. 2011; 35(9):712–21. doi: 10.1016/j.chiabu.2011.05.010 21940046

[35] Eisenberg N, Miller PA. The relation of empathy to prosocial and related behavior. Psychol Bull. 1987 Jan; 101(1):91–119. doi: 10.1037/0033-2909.101.1.91 3562705

[36] Karl A, Williams MJ, Cardy J, Kuyken W, Crane C. Dispositional self-compassion and responses to mood challenge in people at-risk for depressive relapse/recurrence. Clin Psychol Psychother. 2018 Sep; 25 (5):621–633. doi: 10.1002/cpp.2302 29896818 PMC6221037

[37] Piff PK, Dietze P, Feinberg M, Stancato DM, Keltner D. Awe, the small self, and prosocial behavior. J Pers Soc Psychol. 2015; 108(6):883–99. doi: 10.1037/pspi0000018 25984788

[38] Pijper J, de Wied M, van Rijn S, van Goozen S, Swaab H, Meeus W. Executive attention and empathy-related responses in boys with oppositional defiant disorder or conduct disorder, with and without comorbid anxiety disorder. Child Psychiatry Hum Dev. 2018; 49(6):956–965. doi: 10.1007/s10578-018-0810-z 29752662 PMC6208975

[39] Goubert L, Vervoot T, Sullivan MJL, Verhoeven K, Crombez G. Parental emotional responses to their child’s pain: The role of dispositional empathy and catastrophizing about their child’s pain. J Pain. 2008; 9(3): 272–9. doi: 10.1016/j.jpain.2007.11.006 18206424

[40] Rodriguez CM. Analog of parental empathy: Association with physical child abuse risk and punishment intentions. Child Abuse Negl. 2013; 37(8):493–9. doi: 10.1016/j.chiabu.2012.10.004 23294605

[41] Rodriguez CM, Cook AE, Jedrziewski CT. Reading between the lines: Implicit assessment of the association of parental attributions and empathy with abuse risk. Child Abuse Negl. 2012; 36(7–8):564–71. doi: 10.1016/j.chiabu.2012.05.004 22858063

[42] Watson D, Clark LA. The PANAS-X: The manual for the Positive and Negative Affect Schedule-Expanded Form. University of Iowa; 1994.

[43] Wakabayashi A, Baron-Cohen S, Wheelwright S, Goldenfield N, Delaney J, Fine D, et al. Development of short forms of the Empathy Quotient (EQ-Short) and the Systemizing Quotient (SQ-Short). Pers Individ Dif. 2006; 41(5):929–940. doi: 10.1016/j.paid.2006.03.017

[44] Davis MH. Measuring individual differences in empathy: A multi-dimensional approach. J Pers Soc Psychol. 1983; 44(1):113–126. doi: 10.1037/0022-3514.44.1.113

[45] Vachon DD, Lynam DR. Fixing the problem with empathy: Development and validation of the affective and cognitive measure of empathy. Assessment. 2016; 23(2):135–49. doi: 10.1177/1073191114567941 25612628

[46] Nunnally JC, Bernstein IH. Psychometric theory, 3rd ed. New York (NY): McGraw Hill; 1994.

[47] Gonzalez S, Rodriguez CM. Parental Empathy: Assessing parents’ ability to understand children’s perspectives. Paper presented at the annual meeting of the Southeastern Psychological Association. 2020, April.

[48] Reitman D, Rhode PC, Hupp SDA, Altobello C. Development and validation of the Parental Authority Questionnaire—Revised. J Psychopathol Behav Assess. 2002; 24:119–127. doi: 10.1023/A:1015344909518

[49] McEachern AD, Dishion TJ, Weaver CM, Shaw DS, Wilson MN, Gardner F. Parenting Young Children (PARYC): Validation of a self-report parenting measure. J Child Fam Stud. 2012; 21(3):498–511. doi: 10.1007/s10826-011-9503-y 22876108 PMC3412343

[50] Rodriguez CM. Parental discipline reactions to child noncompliance and compliance: Association with parent-child aggression indicators. J Child Fam Stud. 2016; 25(4):1363–1374. doi: 10.1007/s10826-015-0308-2

[51] Rutherford HJV, Crowley MJ, Gao L, Francis B, Schultheis A, Mayes LC. Prenatal neural responses to infant faces predict postpartum reflective functioning. Infant Behav Dev. 2018; 53:43–48. doi: 10.1016/j.infbeh.2018.09.003 30314716

[52] Ortner CNM, De Koning M. Effects of regulating positive emotions through reappraisal and suppression on verbal and non-verbal recognition memory. PloS One. 2013; 8(4):e62750. doi: 10.1371/journal.pone.0062750 23658647 PMC3637263

[53] Park JL, Johnston C, Colalillo S, Williamson D. Parents’ attributions for negative and positive child behavior in relation to parenting and child problems. J Clin Child Adolesc Psychol. 2018; 47(Sup 1):S63–S75. doi: 10.1080/15374416.2016.1144191 27070717

[54] Britton JC, Phan KL, Taylor SF, Fig LM, Liberzon I. Corticolimbic blood flow in posttraumatic stress disorder during script-driven imagery. Biol Psychiatry. 2005; 57(8):832–40. doi: 10.1016/j.biopsych.2004.12.025 15820703

